# The technical reliability and biotemporal stability of cerebrospinal fluid biomarkers for profiling multiple pathophysiologies in Alzheimer’s disease

**DOI:** 10.1371/journal.pone.0193707

**Published:** 2018-03-05

**Authors:** Bianca A. Trombetta, Becky C. Carlyle, Aaron M. Koenig, Leslie M. Shaw, John Q. Trojanowski, David A. Wolk, Joseph J. Locascio, Steven E. Arnold

**Affiliations:** 1 MGH Institute for Neurodegenerative Disease, Department of Neurology, Massachusetts General Hospital, Harvard Medical School, Charlestown, MA, United States of America; 2 Center for Neurodegenerative Disease Research, Department of Pathology and Laboratory Medicine, Perelman School of Medicine of the University of Pennsylvania, Philadelphia, PA, United States of America; 3 Penn Memory Center, Department of Neurology, Perelman School of Medicine of the University of Pennsylvania, Philadelphia, PA, United States of America; Nathan S Kline Institute, UNITED STATES

## Abstract

**Objective:**

Alzheimer’s disease (AD) is a complex neurodegenerative disease driven by multiple interacting pathophysiological processes that ultimately results in synaptic loss, neuronal death, and dementia. We implemented a fit-for-purpose modeled approach to qualify a broad selection of commercially available immunoassays and evaluate the biotemporal stability of analytes across five pathophysiological domains of interest in AD, including core amyloid-β (Aβ) and tau AD biomarkers, neurodegeneration, inflammation/immune modulation, neurovascular injury, and metabolism/oxidative stress.

**Methods:**

Paired baseline and eight-week CSFs from twenty participants in a clinical drug trial for mild cognitive impairment (MCI) or mild dementia due to AD were used to evaluate sensitivity, intra-assay precision, inter-assay replicability, and eight-week biotemporal stability for sixty unique analytes measured with commercially available single- and multi-plex ELISA assays. Coefficients of variation (CV) were calculated, and intraclass correlation and Wilcoxon signed rank tests were applied.

**Results:**

We identified 32 biomarker candidates with good to excellent performance characteristics according to assay technical performance and CSF analyte biotemporal stability cut-off criteria. These included: 1) the core AD biomarkers Aβ_1–42_, Aβ_1–40_, Aβ_1–38_, and total tau; 2) non-Aβ, non-tau neurodegeneration markers NfL and FABP3; 3) inflammation/immune modulation markers IL-6, IL-7, IL-8, IL-12/23p40, IL-15, IL-16, MCP-1, MDC, MIP-1β, and YKL-40; 4) neurovascular markers Flt-1, ICAM-1, MMP-1, MMP-2, MMP-3, MMP-10, PlGF, VCAM-1, VEGF, VEGF-C, and VEGF-D; and 5) metabolism/oxidative stress markers 24-OHC, adiponectin, leptin, soluble insulin receptor, and 8-OHdG.

**Conclusions:**

Assays for these CSF analytes demonstrate consistent sensitivity, reliability, and biotemporally stability for use in a multiple pathophysiological CSF biomarker panel to profile AD. Their qualification enables further investigation for use in AD diagnosis, staging and progression, disease mechanism profiling, and clinical trials.

## Introduction

Alzheimer’s disease (AD) and related disorders (including Lewy body dementias, frontotemporal lobar degenerations, and vascular cognitive impairment and dementias) are complex neurodegenerative diseases driven by vicious pathophysiological cycles of protein misfolding [1], inflammation [2,3], neurovascular dysfunction [4], oxidative injury [5,6], and disruption of metabolic pathways [7,8]. To varying degrees, these promote excitotoxicity [9], autophagy [10], apoptosis [11], and necrosis [12], with resultant synaptic loss and neuronal death [13,14]. Progressive dementia of insidious onset is the clinical manifestation of these processes that evolve over years to decades prior to expression of clinical symptoms [15]. While the core amyloid-β (Aβ) and tau biomarkers of AD [16,17] are highly associated with the presence of signature plaque and tangle pathological hallmarks of AD in the brain [16], they do not assess other fundamental biochemical aspects of the disease, such as non-amyloid and non-tau neurodegeneration or metabolic, immune, and neurovascular dysfunction [18–21]. A panel of robust biomarkers that directly reflect these concomitant pathophysiologies may prove a better indicator of disease diagnosis [22,23], subtypes, staging, and activity, as well as treatment-specific target engagement.

As clinical trials continue to move towards pre-symptomatic individuals and disease prevention, it is critical to establish biofluid markers that may sensitively detect biochemical changes associated with disease onset and the intersecting pathophysiologies that drive disease progression [24,25]. A panel of analytes that represent multiple facets of AD pathology and pathophysiology, as opposed to a single marker, may offer increased specificity and sensitivity in diagnosis [22,23], but more importantly, may also profile AD subtypes to enable precision medicine. Current clinical trials for AD focus on disease modification with investigational new agents that target general neuroprotection, neuroinflammation, metabolic and oxidative dysfunction, and neurovascular injury. Determining how active each of these pathophysiologies are in a given patient with pathway-oriented biomarkers may help guide choice of therapy, and would also be important for demonstrating and monitoring the effects of pharmacodynamic target engagement.

A key consideration for the development of any biomarker is the reproducibility of results within and across times and laboratories. While the core AD biomarkers have been extensively evaluated in terms of precision, pre-analytical factors affecting measures, and utility in clinical research and care [21,26–29], validations for newer candidate cerebrospinal fluid (CSF) biomarkers are still in an early phase. Of particular importance for clinical trials is confidence in the stability of biomarkers over short-term repeat collections from the same individual, independent of disease-related pathophysiological changes [30,31]. Highly dynamic analytes whose levels fluctuate widely from day to day as a result of diet, restless sleep, or other biorhythms or environmental influences, would not prove reliable in a clinical trial where a limited number of samples are collected over long periods of time. Like technical precision of an assay, this "biotemporal stability" of a biomarker needs to be considered in sample size determinations and study design so the measurements reflect true disease progression or drug effect as opposed to random noise. Low baseline variability over brief periods of time, as has been established with the core AD biomarkers [32–36], increases the value of a CSF biomarker as a routine measurement in a clinical setting, suggesting that it may be sensitive enough to detect disease-related differences, disease progression over longer periods of time, or biochemical changes in response to intervention [31]. While there are a few reports describing short-term intra-individual variation of AD-relevant biomarkers in serum [31,37] and over longer intervals (annual) in CSF [35], stability over shorter intervals in CSF is rarely investigated or reported. Opportunities to conduct such analyses are scarce, as multiple lumbar punctures within short timeframes are seldom performed, even in the context of clinical research.

We implemented a fit-for-purpose modeled approach for evaluating and qualifying a broad selection of commercially available immunoassays for exploratory use with CSF. The assays represent five key domains of AD pathophysiology: 1) core AD amyloid-β and total tau biomarkers, 2) non-Aβ and non-tau neurodegeneration, 3) inflammation and immune modulation, 4) neurovascular markers, and 5) metabolism and oxidative stress. We identified 32 biomarker candidates with excellent performance characteristics by assessing technical assay reliability and biotemporal intra-individual variation of each biomarker in CSFs collected at two timepoints over an eight-week interval from individuals with mild cognitive impairment (MCI) or mild dementia due to AD. These candidate analytes may be used to develop a broad, practical biomarker panel that simultaneously portrays the diverse pathophysiological processes involved in AD and related disorders. These rigorously validated analytes should perform consistently and reliably in profiling the complex pathophysiology of AD and monitoring changes during disease progression and intervention, ultimately enabling clinical trials and allowing personalized treatment.

## Materials and methods

### Study participants

CSFs were obtained at baseline and after 8 weeks as part of a pilot randomized placebo-controlled clinical trial investigating the effects of the drug metformin (versus placebo) in MCI/AD (NCT01965756) [38]. All subjects provided written informed consent for participation and use of CSF in future research in Human Subjects Institutional Review Board (IRB)-approved protocols at the University of Pennsylvania. All subjects had a clinical diagnosis of amnestic MCI or mild dementia due to AD. Demographic and clinical characteristics are presented in **Table 1**. As previously described [38], eligibility criteria included: age 55 to 80 years at screening, clinical diagnosis of MCI or mild dementia due to AD with a Clinical Dementia Rating [39] global ≤ 1.0, Mini-Mental State Examination [40] > 19, Geriatric Depression Scale [41] total < 6 to exclude concomitant depression, Modified Hachinski Ischemic Scale [42] score < 4 to exclude subjects with potential vascular etiology to their cognitive complaints, fasting blood glucose < 110 or HgbA1c < 6.0 to exclude subjects with diabetes or prediabetes, and at least one positive biomarker consistent with AD (i.e. previous Aβ CSF, fluorodeoxyglucose or Aβ positron emission tomography, or volumetric MRI). Individuals on an acetylcholinesterase inhibitor were required to be on a stable dose for at least 2 months prior to screening. Based on prerequisites for the intervention under investigation, potential subjects were ineligible if they had past or current diabetes or renal disease, evidence of infarcts, focal intracranial lesions or other neurodegenerative conditions, or unstable medical or psychiatric illness. A total of 20 participants were enrolled: 9 women and 11 men, with a mean age of 70.1 years. *APOE* ε4 carrier status was inferred following the conclusion of the study using a validated immunoassay (K4699, BioVision) to measure Apolipoprotein E (ApoE) ε4 in plasma according to manufacturer’s instructions.

**Table 1:** Baseline demographic and clinical characteristics of study participants.

**Demographic and *APOE* characteristics at baseline**		**Mean (SD)**
	Age, years	70.10 (6.89)
	Sex, female, n (%)	9 (45%)
	Education, years	16.70 (2.77)
	*ApoE* ε4 positive, n (%)	13 (65%)
**Clinical characteristics**		
	CDR Sum of Boxes	2.4 (1.1)
	CDR-Global, median (range)	0.5 (0.5–1)
	MMSE	25.9 (2.3)
	DSRS	9.10 (4.29)
	GDS	1.20 (1.15)

Characteristics presented as mean (SD) unless stated otherwise. *ApoE* ε4 positive: *ApoE* phenotype has at least one ε4 allele. Abbreviations: SD, standard deviation; CDR, Clinical Dementia Rating; MMSE, Mini-Mental State Examination; DSRS, Dementia Severity Rating Scale; GDS, Geriatric Depression Scale. *N* = 20.

### Cerebrospinal fluid collection and measurement overview

CSF samples were collected by lumbar puncture (LP) at baseline and again at 8 weeks with adherence to ADNI protocol (http://www.adni-info.org/). The procedure was performed between 8 a.m. and 10 a.m. in all participants to minimize effects of diurnal variation. Using 24-gauge Sprotte^®^ atraumatic needles, 20 mL of CSF was collected into polypropylene syringes according to standard procedure. Within 30 minutes of collection, CSF was aliquoted into 0.5 mL polypropylene tubes, bar-coded, frozen, and stored at -80°C for subsequent analysis.

CSFs were tested in duplicate in each of three experimental blocks to assess the intra- and inter-plate reliability of candidate assays. Repeat CSFs from 9 subjects in the 8-week placebo arm were used in paired assays and tested in each block to determine short-term intra-individual variation. After initial data inspection, samples from one participant in the metformin group were disregarded due to hemolytic contamination, resulting in artificially elevated analyte concentrations. Assays were conducted in the Arnold Lab at the Massachusetts General Hospital Institute for Neurodegenerative Diseases (MIND). In addition, amyloid-β peptide 1–42 (Aβ_1–42_), total tau (tTau), and phospho-tau (pTau) data were available from prior testing in these samples at the ADNI Biomarker Core / Shaw Lab at the University of Pennsylvania [38].

### Biochemical procedures

The performance characteristics of 60 unique potential biomarker analytes were evaluated in twenty single or multi-plexed panel kits (**Table 2**). CSF concentrations of 54 analytes were examined using the Meso-Scale Discovery (MSD) platform with commercially available simplex and multi-plex electrochemiluminescent (ECL) immunoassays (Meso-Scale Diagnostics, LLC, Rockville, MD). Along with replicating the core AD biomarkers Aβ_1–42_ and tTau, we tested other potential analytes in the pathophysiological domains of neurodegeneration, metabolism and oxidative damage, neurovascular injury, and inflammation/immune modulation. Fourteen MSD assay panels for biomarkers of interest were chosen based on a number of factors including *a priori* pathophysiological relevance, assay availability, previous assay use in the literature, specific reported features of concentration sensitivity and range, previous use in CSF (if any) and previous findings in AD. An additional 6 colorimetric ELISA kits were used to measure markers not available through MSD. All kits were purchased in bulk to minimize lot-to-lot variability.

**Table 2:** Evaluated immunoassay kits, including assay characteristics and CSF dilutions.

Commercial panel name		Company	Catalog number	Biomarker targets	CSF Dilution
**Core AD assays**					
	V-PLEX Plus Human Total Tau	MSD	K151LAG	total tau	1:4
	V-PLEX Plus Aβ Peptide Panel 1 (6E10)	MSD	K15200G	Aβ_1–38_, Aβ_1–40_, Aβ_1–42_	1:2
**Aβ and tau-independent neurodegeneration assays**					
	U-PLEX Plus Human α-Synuclein	MSD	K151WKP	α-Synuclein	1:8
	Human FABP3	MSD	K151HTD	FABP3	Neat^†^
	NfL (Neurofilament-light) ELISA	UmanDiagnostics	10–7002	Neurofilament-light	1:2
**Metabolic assays**					
	24(S)-Hydroxycholesterol ELISA	Enzo Life Sciences	ADI-900-210-0001	24-OHC	1:2
	Human Active GLP-1 (7–36) amide, Insulin, Glucagon, Leptin	MSD	K15173C	Active GLP-1 (7–36) amide, Insulin, Glucagon, Leptin	Neat^†^
	Human Adiponectin	MSD	K151BXC	Adiponectin	1:10
	Insulin Receptor Human ELISA	BioVendor	RD1991041200R	Soluble Insulin Receptor	1:2^†^
	VGF Nerve Growth Factor Inducible (VGF) Human ELISA	Hölzel Diagnostika	SEB166Hu	VGF	Neat^†^
**Oxidative stress assays**					
	OxiSelect CML Competitive ELISA	Cell Biolabs, Inc.	STA-816	CML	Neat^†^
	OxiSelect Oxidative DNA Damage ELISA	Cell Biolabs, Inc.	STA-320	8-OHdG	Neat^†^
**Neuroinflammatory and vascular injury assays**					
	V-PLEX Plus Angiogenesis panel 1 (human)	MSD	K15190G	FGF (basic), Flt-1, PlGF, Tie-2, VEGF-C, VEGF-D	Neat^†^
	V-PLEX Plus Chemokine Panel 1 (human)	MSD	K15047G	Eotaxin, Eotaxin-3, IP-10, MCP-1, MCP-4, MDC, MIP-1α, MIP-1β, TARC	Neat^†^
	V-PLEX Plus Cytokine panel 1 (human)	MSD	K15050G	IL-1α, IL-5, IL-7, IL-12/IL-23p40, IL-15, IL-16, IL-17A, TNF-β, VEGF	Neat^†^
	Human MMP 2-Plex Ultra-Sensitive Panel	MSD	K15033C	MMP-2, MMP-10	Neat^†^
	Human MMP 3-Plex Ultra-Sensitive Panel	MSD	K15034C	MMP-1, MMP-3, MMP-9	Neat^†^
	V-PLEX Plus Proinflammatory Panel 1 (human)	MSD	K15049G	IFN-ɣ, IL-1β, IL-2, IL-4, IL-6, IL-8, IL-10, IL-13, TNF-α	Neat^†^
	V-PLEX Plus Vascular Injury Panel 2 (human)	MSD	K15198G	CRP, ICAM-1, SAA, VCAM-1	1:5
	Human YKL-40	MSD	K151NHD	YKL-40	1:50^†^

^†^ Datasheet only included published dilution factors for serum and plasma. Dilution factor required for CSF was determined experimentally. Abbreviations: MSD, Meso Scale Diagnostics, LLC.

Assays were performed according to manufacturer’s specifications and samples diluted per assay requirements. For kits commercially validated for use with CSF, samples were diluted according to published dilution recommendations. These included: V-PLEX Plus Human Total Tau, V-PLEX Plus Aβ Peptide Panel 1 (6E10), U-PLEX Plus Human α-Synuclein, V-PLEX Plus Vascular Injury Panel 2, and NF-light (NfL) ELISA. Recommended dilutions ranged from 1:2–1:8. The remaining 15 kits did not include published dilution recommendations for CSF samples, necessitating experimental determination of the minimum required dilution (MRD) prior to final analysis (designated with a † in **Table 2**). For many of these assays, CSF samples were measured neat to allow concentrations to fall within a detectable range.

**Fig 1:**
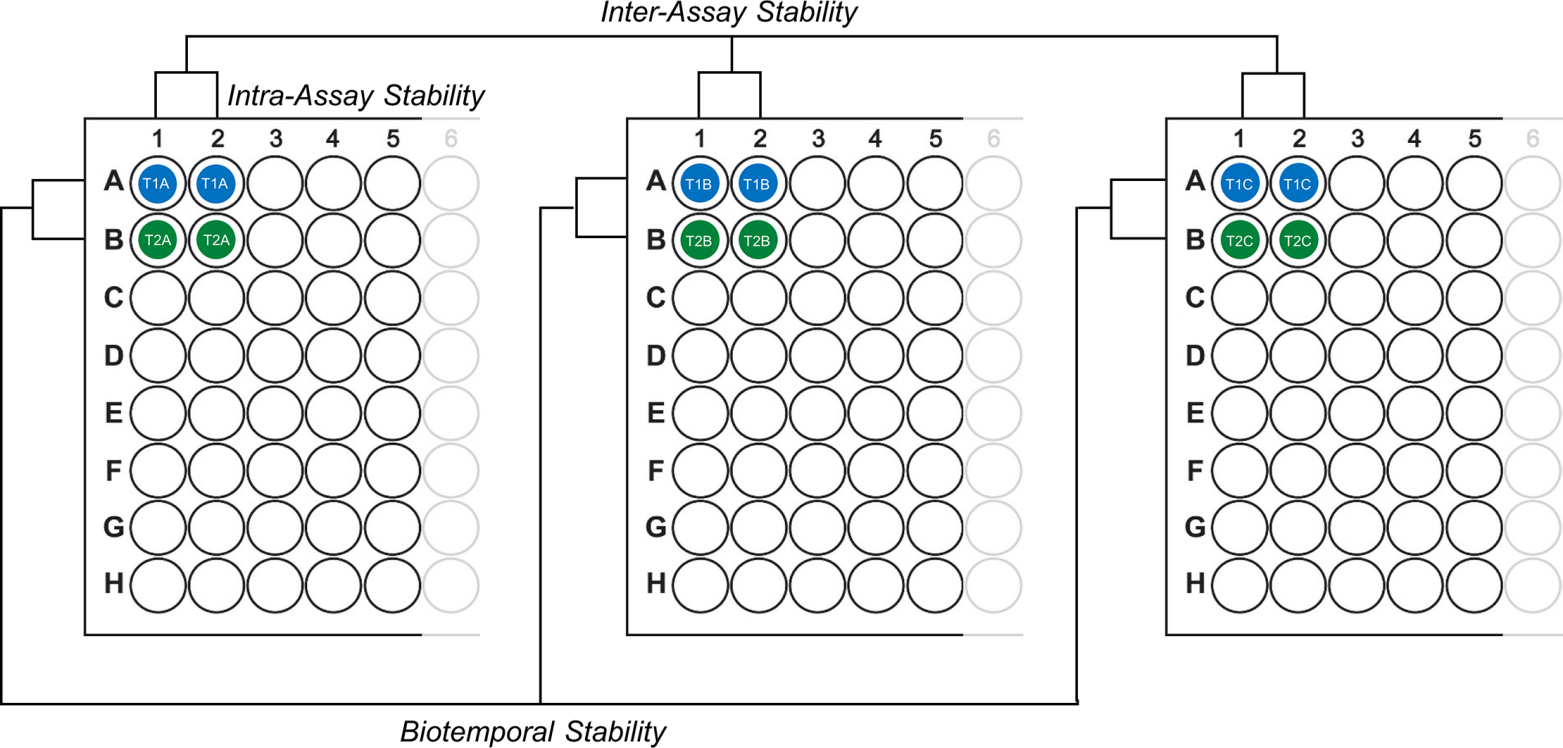
Structure of the assay stability evaluation scheme. Duplicate samples were located on individual plates as shown, to allow for assessment of intra- and inter-assay precision and biotemporal stability of analyte measures. T1 and T2 denote repeat-collected CSFs from the same individual; A-C indicate different aliquots of the same CSF sample. *N* = 35 samples were included on each individual plate, in addition to a standard curve and spiked controls.

The plate design scheme for sample wells and reliability measures is shown in **Fig 1**. Each plate contained the entire cohort of CSF samples, and all paired CSF samples per subject were included on the same assay in adjacent wells. This design ensured that plate-to-plate variability would not compromise biotemporal analysis of analyte concentrations between paired repeat-collection CSFs. Each CSF sample was assayed in duplicate per plate, and each plate experiment was replicated three times. A different 0.5 mL aliquot of sample was used for each assay replicate. Serially diluted standard curves and spiked controls were included on every plate and measured in duplicate.

Numerical data obtained on the MSD platform were generated using the MSD Discovery Workbench® 4.0 software. For colorimetric ELISA kits, absorbance values were collected using a microplate reader (Victor 2 Multi-Label Microplate Reader, Perkin Elmer-Wallac) and sample concentrations manually calculated against the standard curve using a 4-Parameter Logistic Regression.

### Statistical analysis

Statistical analyses were conducted using SPSS Statistics 24.0 (IBM Corp., Armonk, N.Y., USA), R 3.3.2 [43], and RStudio 1.0.136 [44]. CSF analyte concentrations were natural-logarithm transformed prior to computing relevant statistical tests that required normally distributed residuals.

A number of assays examined here had not been reported previously in CSF. Consistent detection of an analyte in plasma does not ensure detection in CSF, as the protein may not be generated in the CNS, its levels may be altered by blood brain barrier (BBB) selectivity, and/or its detection may be compromised by matrix effects. To verify whether each assay had sufficient sensitivity to detect an analyte(s) in CSF, we compared sample concentrations of analytes to the assay’s lower limit of detection (LLOD), specific to each individual plate and analyte. The LLOD was calculated by the Discovery Workbench 4.0 software as 2.5 standard deviations above the background signal. For colorimetric assays, the published LLOD was used as the assay’s sensitivity threshold. A biomarker assay was considered to be satisfactorily sensitive if it was consistently measured above the assay LLOD in > 80% of samples across all plates.

For use in any comparative study, it is critical that repeat measures are technically reliable. Moreover, to detect change with treatment in the context of typically slow disease progression, CSF levels should be biologically consistent and not fluctuate widely over short periods of time. An experimental scheme was designed to assess all forms of assay stability for analytes that passed LLOD criteria: intra-assay, inter-assay, and biotemporal stability (**Fig 1**).

Intra-assay reliability was assessed by calculating the median coefficient of variation (CV), or relative standard deviation, for duplicate sample concentrations within each of the 3 plates. Inter-assay reliability was assessed by calculating CV values for duplicate samples across three experimental blocks and tested statistically using intra-class correlation coefficients (ICCs) [45,46]. ICC estimates and 95% Confidence Intervals (CIs) were calculated using SPSS Statistics 24.0 based on ICC(3,3), a mean-rating, absolute agreement, 2-way mixed-effects model (recommended in [47]). Mean normalization was first applied to each plate to correct for day-to-day technical variation, before calculation of the CV. Analytes with both intra- and inter-assay CVs below 15% were considered to have acceptable technical reliability. Data were natural-logarithm transformed prior to ICC analysis. ICCs were interpreted as follows: ICCs > 0.75 indicated strong reliability, ICCs between 0.5–0.75 reflected moderate to good reliability, and ICCs < 0.5 indicated poor reliability [48].

Samples from subjects in the placebo group were used for the intra-individual biotemporal variation assessment of each analyte that passed the technical precision criteria. Paired CSFs collected at baseline and after the first treatment block (8 weeks apart) were used to calculate CV values representing the biotemporal stability of analytes. The mean concentrations for each sample across the three experimental blocks was used for analysis. ICCs were not calculated for biotemporal variation as the sample size was not sufficient to satisfy test conditions. Similarly, given that analyte distribution could not be assumed to be normal, a Wilcoxon Signed-Ranks Test was used to assess whether there were significant differences between CSF collection timepoints. Finally, an analyte with a calculated biotemporal CV below 15% was considered to have low baseline variability.

## Results

### Assay sensitivity for analytes in CSF

A total of 35 CSF samples collected from twenty study participants were included in this analysis. Five samples were unavailable due to an unsuccessful lumbar puncture or subject refusal. Assay sensitivity was assessed for twenty single-plex and multi-plex assays used in this study (**Table 2**). Percentages of sample measurements with concentrations falling above the LLOD were calculated using concentrations from all sample replicates across the three experimental blocks. In total, 105 measurements per biomarker were taken into consideration to determine whether the target protein could be reliably detected and measured in CSF (**S1 Table and S1 Fig**).

For the four core AD biomarkers (tTau, Aβ_1–38_, Aβ_1–40_, and Aβ_1–42_) and the three Aβ and tau-independent "neurodegeneration" markers neurofilament light-chain (NfL), α-Synuclein, and fatty acid binding protein 3 (FABP3), all 105 sample concentrations were measurable well above the LLOD, as expected.

Of the 30 inflammatory/immune markers, sixteen were measurable in all samples (eotaxin-3, IP-10, MCP-1, MDC, MIP-1α, MIP-1β, TARC, IL-7, IL-12/IL-23p40, IL-15, IL-16, IL-6, IL-8, SAA, CRP, and YKL-40). IL-5, IL-10, and eotaxin were measurable in > 90% of samples (96/105, 101/105, and 101/105, respectively). IFN-ɣ was measurable in 89.52% (94/105) of CSF samples, and TNF-α was measurable in 80.95% (85/105). The remaining nine inflammatory markers (MCP-4, IL-17A, IL-1α, TNF-β, IL-1β, IL-2, IL-4, IL-13, and Tie-2) did not meet assay sensitivity criteria, with < 80% of all measured samples falling above the qualifying LLOD threshold.

The thirteen neurovascular biomarkers were considered measurable in CSF, with ten of those analytes measurable in all 105 assayed samples including the matrix metalloproteinases MMP-1, MMP-2, MMP-3, and MMP-10, vascular endothelial growth factor (VEGF) and the VEGR receptor (Flt-1), VEGF-D, vascular cell adhesion molecule-1 (VCAM-1), intercellular adhesion molecule-1 (ICAM-1) and phosphatidylinositol-glycan biosynthesis class F protein (PlGF). The neurovascular markers MMP-9 and VEGF-C were measurable in > 90% of samples (101/105 and 104/105, respectively), and fibroblast growth factor-basic (FGF-basic) was measurable in 89.52% (94/105) of assayed samples.

Four of the ten metabolic markers were measurable in all CSF samples: 24S-hydroxycholesterol (24-OHC), adiponectin, soluble insulin receptor (sIR), and 8-hydroxydeoxy guanosine (8-OHdG). Leptin was measurable in 84.29% (59/70) of included samples. Active glucagon like protein-1 (GLP-1), glucagon, and carboxymethyl lysine (CML) were not detectable in any CSF samples using the applied methods. Insulin was not considered measurable in CSF (only 54.29% of sample concentrations fell above the LLOD), but because of interest in this metabolic hormone in AD [7,8] we pursued it with three alternative commercial assays, though none yielded better results. The VGF nerve growth factor inducible (VGF) immunoassay was not reproducible in our hands and was discontinued (**S1 Table**).

### Technical precision: Intra-assay reliability

Analyses of intra-assay reliability were performed only if biomarker candidates were measurable in > 80% of cases in the initial sensitivity screening. Analytes with marginal concentrations in CSF might be expected to exhibit poor precision and reliability due to their proximity to the LLOD [49]. Therefore, insulin, active GLP-1, glucagon, CML, MCP-4, IL-17A, IL-1α, TNF-β, IL-1β, IL-2, IL-4, IL-13, and Tie-2 were excluded from subsequent analyses.

Forty-two of the remaining 46 analytes met acceptable intra-assay reliability criteria (**S1 Table**). The four core AD biomarkers (tTau, Aβ_1–38_, Aβ_1–40_, and Aβ_1–42_) and three neurodegenerative markers (α-Synuclein, FABP3, and NfL) had excellent intra-assay precision (CVs < 5%). All five metabolic and oxidative stress markers (24-OHC, adiponectin, leptin, sIR, and 8-OHdG) and eleven of the thirteen neurovascular markers (MMP-1, MMP-2, MMP-3, MMP-10, Flt-1, ICAM-1, PlGF, VCAM-1, VEGF, VEGF-C, and VEGF-D) also had intra-assay CVs below 5%. Two neurovascular markers, MMP-9 and FGF (basic), had intra-assay CVs below 10%, which met acceptable intra-assay criteria.

The inflammatory analytes were the only class to include some intra-assay CVs that did not meet predetermined precision criteria. Fifteen of the 21 inflammatory analytes (IP-10, MCP-1, MIP-1β, IL-5, IL-7, IL-12/IL-23p40, IL-15, IL-16, IL-6, IL-8, IL-10, TNF-α, SAA, CRP, and YKL-40) exhibited excellent or acceptable intra-assay precision with CV values below 15%. Two inflammatory candidates (IFN-ɣ and MDC) nearly made the acceptable threshold (15.47% and 15.75%, respectively), and were kept under consideration due to their proximity to the acceptability criteria. Three candidates (eotaxin-3, MIP-1α, and TARC) had intra-assay CVs between 16–20%, and one candidate, eotaxin, had poor intra-assay precision (27.35%). These four analytes were excluded from further analyses.

### Technical replicability: Inter-assay reliability

Inter-assay reliability was assessed using ICC(3,3) taking into account the mean concentrations of each sample (*n* = 35) measured across three experimental blocks. The overall inter-assay CV for each biomarker candidate was evaluated by calculating the CV between all technical replicates across the three experimental blocks after applying a mean-normalization adjustment to sample concentrations on repeated assays. Both analytical methods were used to stringently identify candidates with the best overall inter-assay replicability (i.e. exhibiting both ICC reliability > 0.5 and an inter-assay CV < 15%). Failure to meet performance criteria under either analysis served as an indication of poor inter-assay replicability.

All ICC(3,3) coefficients for the 42 biomarker candidates still in consideration were significant (*p* < 0.05). Thirty-five of these analytes demonstrated acceptable inter-assay reliability (**S2 Table**).

As anticipated, the four core AD biomarkers (tTau, Aβ_1–38_, Aβ_1–40_, and Aβ_1–42_) exhibited both strong inter-assay reliability (ICC > 0.75) and inter-assay CVs below 15%. Likewise, all five metabolic markers (adiponectin, leptin, sIR, 8-OHdG, and 24-OHC) exhibited strong inter-assay reliability (ICC > 0.75) and inter-assay CVs below 15%. Two of the three neurodegenerative markers (FABP3 and NfL) met acceptable inter-assay criteria under both analyses (ICC > 0.5 and CV < 15%). Although α-Synuclein had an ICC of 0.8 (indicating strong reliability when transformed to a normally-distributed dataset), it did not meet acceptable inter-assay CV criteria (CV: 16.76%) and was discounted.

Of the thirteen neurovascular markers, two analytes (MMP-9 and FGF (basic)) were excluded based on poor inter-assay CVs (19.25% and 23.94%, respectively) despite having ICCs > 0.75. The remaining eleven neurovascular markers (MMP-1, MMP-2, MMP-3, MMP-10, Flt-1, ICAM-1, PlGF, VCAM-1, VEGF, VEGF-C, and VEGF-D) were classified as having strong inter-assay reliability under the combined analysis (ICC > 0.75, CV < 15%).

Ten of the seventeen inflammatory markers (MCP-1, MIP-1β, IL-7, IL-12/IL-23p40, IL-6, IL-8, IL-10, SAA, CRP, and YKL-40) had acceptable CV values (< 15%) in addition to strong inter-assay reliability (ICC > 0.75). An additional three inflammatory analytes (MDC, IL-15, and IL-16) exhibited moderate inter-assay reliability (ICC: 0.5–0.75, CV < 15%). The remaining four inflammatory candidates (IFN- ɣ, IP-10, IL-5, and TNF-α) were excluded based on either poor ICC reliability (ICC < 0.5) or failure to meet acceptable inter-assay CV criteria of < 15%.

### Biotemporal intra-individual variation

**Fig 2:**
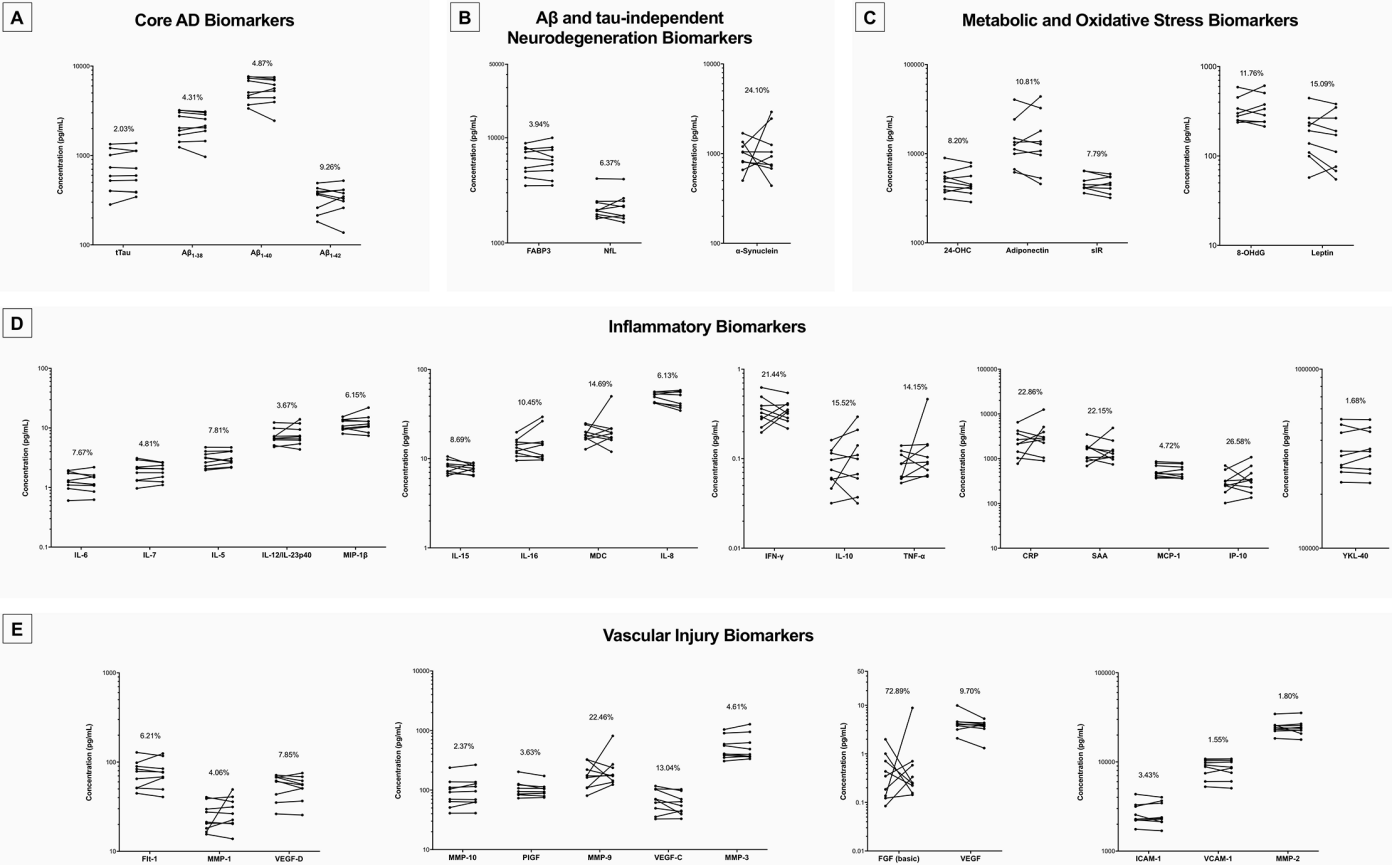
Short-term biotemporal stability of measurable and technically precise analytes in CSF. Biotemporal variation between paired samples over an 8-week interval for (a) core AD biomarkers, (b) Aβ and tau-independent markers of neurodegeneration, (c) metabolic and oxidative stress markers, (d) markers of inflammation and inflammatory modulators, and (e) vascular injury markers. Samples plotted for each analyte, with baseline concentrations connected to corresponding paired week 8 concentrations. Plotted using a log scale, all units converted to pg/mL. *N* = 9 pairs for each analyte, concentrations averaged across three technical replicates. Each analyte plot is labeled with the median biotemporal CV (%). A CV < 15% indicated low intra-individual variation.

The biotemporal variation of each analyte was determined through analysis of mean concentrations for paired CSFs collected from subjects in the placebo group (*n* = 9) at baseline and again eight weeks later after the first treatment block (**Fig 2 and Table 3**). This analysis was performed for the 35 analytes that passed the assay sensitivity, intra-assay and inter-assay precision screenings and remained under consideration as promising candidates. As testing distributions for normality suggested that normality could not be assumed, a Wilcoxon Signed-Ranks Test was used to evaluate whether there were any statistically significant observed differences between paired CSFs. None of the 35 analytes examined showed statistically significant differences between repeat-collected samples (*p* > 0.05), however three exceeded our CV < 15% criterion for acceptable biotemporal stability, including SAA, CRP, and IL-10. The remaining 32 met acceptable biotemporal stability criteria (CV < 15%), including the four core AD markers (tTau, Aβ_1–38_, Aβ_1–40_, and Aβ_1–42_), the two remaining neurodegenerative markers (NfL and FABP3), and the five metabolic markers (24-OHC, adiponectin, leptin, soluble IR, and 8-OHdG). All eleven neurovascular markers that met inter-assay reliability criteria (MMP-1, MMP-2, MMP-3, MMP-10, Flt-1, ICAM-1, PlGF, VCAM-1, VEGF, VEGF-C, and VEGF-D) also exhibited acceptable biotemporal stability (CV < 15%), as did ten of the remaining thirteen inflammation markers (MCP-1, MDC, MIP-1β, IL-7, IL-12/23p40, IL-15, IL-16, IL-6, IL-8, and YKL-40).

**Table 3:** Intra-individual biostability of analyte measurements between repeat-collected CSFs.

	Analyte	Mean baseline concentration (SD)	Mean follow-up concentration (SD)	Median biotemporal CV, % (IQR)	Exact p-value (2-tailed)
**Core AD biomarkers**					
	**Aβ**_**1–38**_ **(pg/mL)**	**2274.36 (780.95)**	**2221.92 (727.66)**	**4.31 (2.39–7.22)**	**0.496**
	**Aβ**_**1–40**_ **(pg/mL)**	**5625.73 (1724.57)**	**5503.12 (1664.84)**	**4.87 (2.86–7.30)**	**0.570**
	**Aβ**_**1–42**_ **(pg/mL)**	**343.35 (102.86)**	**345.14 (108.33)**	**9.26 (6.02–13.75)**	**0.820**
	**Total Tau (pg/mL)**	**721.38 (380.45)**	**732.71 (382.84)**	**2.03 (1.58–5.22)**	**0.820**
**Aβ and tau-independent neurodegeneration biomarkers**					
	α-Synuclein (pg/mL)	1010.23 (365.59)	1244.30 (856.46)	24.10 (13.13–48.79)	0.910
	**FABP3 (ng/mL)**	**6.24 (1.91)**	**6.27 (2.09)**	**3.94 (2.63–5.76)**	**0.652**
	**NfL (pg/mL)**	**2275.26 (728.83)**	**2287.14 (755.59)**	**6.37 (4.51–7.69)**	**0.652**
**Metabolic and oxidative stress biomarkers**					
	**8-OHdG (ng/mL)**^**a**^	**0.34 (0.12)**	**0.40 (0.19)**	**11.76 (8.74–13.84)**	**0.844**
	**24-OHC (ng/mL)**	**5.08 (1.74)**	**4.93 (1.67)**	**8.20 (5.80–10.01)**	**0.734**
	**Adiponectin (ng/mL)**	**15.50 (10.73)**	**16.77 (13.08)**	**10.81 (10.45–25.24)**	**0.734**
	**Leptin (pg/mL)**	**195.67 (116.23)**	**185.28 (122.47)**	**15.09 (10.86–32.37)**	**0.203**
	**Soluble Insulin Receptor (ng/mL)**^**a**^	**4.79 (1.07)**	**4.70 (0.96)**	**7.79 (4.44–9.84)**	**0.383**
**Inflammatory biomarkers**					
	CRP (pg/mL)	2725.01 (1812.81)	3820.46 (3511.84)	22.86 (15.50–42.45)	0.426
	IFN-ɣ (pg/mL)	0.35 (0.13)	0.35 (0.10)	21.44 (14.74–30.04)	1.000
	IL-5 (pg/mL)	3.07 (0.94)	3.16 (0.91)	7.81 (4.76–9.70)	0.359
	**IL-6 (pg/mL)**	**1.33 (0.44)**	**1.29 (0.47)**	**7.67 (5.51–8.82)**	**0.496**
	**IL-7 (pg/mL)**	**1.98 (0.72)**	**1.93 (0.56)**	**4.81 (3.28–9.33)**	**0.652**
	**IL-8 (pg/mL)**	**49.56 (5.70)**	**48.05 (9.84)**	**6.13 (2.90–10.32)**	**0.426**
	IL-10 (pg/mL)	0.09 (0.04)	0.12 (0.09)	15.52 (10.31–44.53)	0.426
	**IL-12/IL-23p40 (pg/mL)**	**7.35 (2.37)**	**8.03 (3.14)**	**3.67 (1.02–8.54)**	**1.000**
	**IL-15 (pg/mL)**	**8.16 (1.39)**	**7.80 (0.94)**	**8.69 (5.59–12.23)**	**0.426**
	**IL-16 (pg/mL)**	**13.66 (3.16)**	**15.71 (7.27)**	**10.45 (2.90–13.90)**	**0.570**
	IP-10 (pg/mL)	328.05 (189.96)	417.52 (300.40)	26.58 (19.69–53.60)	0.426
	**MCP-1 (pg/mL)**	**552.04 (184.84)**	**527.03 (179.64)**	**4.72 (2.44–9.43)**	**0.129**
	**MDC (pg/mL)**	**18.83 (3.75)**	**21.63 (11.05)**	**14.69 (10.44–21.62)**	**1.000**
	**MIP-1β (pg/mL)**	**11.55 (2.51)**	**12.38 (4.28)**	**6.15 (5.55–8.06)**	**0.359**
	TNF-α (pg/mL)	0.09 (0.03)	0.14 (0.13)	14.15 (2.74–27.08)	0.426
	SAA (pg/mL)	1580.41 (840.94)	1738.68 (1282.62)	22.15 (15.25–45.54)	0.652
	**YKL-40 (pg/mL)**	**356214.83 (104425.28)**	**360437.47 (100814.79)**	**1.68 (0.60–6.30)**	**0.910**
**Neurovascular biomarkers**					
	FGF (basic) (pg/mL)	0.56 (0.62)	1.27 (2.84)	72.89 (48.10–103.55)	1.000
	**Flt-1 (pg/mL)**	**76.82 (27.02)**	**78.47 (27.88)**	**6.21 (3.45–6.59)**	**0.820**
	**ICAM-1 (pg/mL)**	**2681.35 (791.79)**	**2678.38 (815.11)**	**3.43 (2.76–5.73)**	**0.910**
	**MMP-1 (pg/mL)**	**25.42 (0.37)**	**29.07 (11.35)**	**4.06 (3.19–8.44)**	**0.570**
	**MMP-2 (pg/mL)**	**24707.42 (4339.36)**	**24440.73 (4896.25)**	**1.80 (1.19–3.40)**	**0.910**
	**MMP-3 (pg/mL)**	**549.29 (258.35)**	**574.36 (328.08)**	**4.61 (3.55–6.81)**	**0.652**
	MMP-9 (pg/mL)	186.74 (89.13)	249.82 (215.74)	22.46 (14.81–54.49)	0.734
	**MMP-10 (pg/mL)**	**100.85 (59.74)**	**108.39 (67.40)**	**2.37 (0.73–7.95)**	**0.129**
	**PlGF (pg/mL)**	**108.86 (39.56)**	**102.45 (29.22)**	**3.63 (2.00–5.33)**	**0.301**
	**VCAM-1 (pg/mL)**	**8709.32 (2019.20)**	**8657.81 (2088.87)**	**1.55 (0.65–4.15)**	**1.000**
	**VEGF (pg/mL)**	**4.48 (2.18)**	**3.76 (1.07)**	**9.70 (3.47–16.16)**	**0.098**
	**VEGF-C (pg/mL)**	**71.45 (30.62)**	**61.10 (25.06)**	**13.04 (2.82–17.93)**	**0.129**
	**VEGF-D (pg/mL)**	**55.89 (16.59)**	**53.49 (15.27)**	**7.85 (3.00–10.93)**	**0.359**

Analytes that met both inter-assay and biotemporal stability highlighted in bold. Mean concentration across 3 plate replicates used to assess biotemporal stability. Mean normalized data of paired CSFs collected 8 weeks apart were used to calculate biotemporal CVs. A CV < 15% indicated low intra-individual variation for a given analyte. Abbreviations: SD, standard deviation; CV, coefficient of variation; IQR, inter-quartile range.

^a^ *N* = 9 except for 8-OHdG and sIR, where only 8 paired samples were available due to sample volume restrictions.

Raw data for all analytes included in the study are provided in **S3 Table**.

## Discussion

We performed comprehensive fit-for-purpose testing of commercially available immunoassays in CSF and identified a broad selection of reliably measured, biotemporally stable markers that represent multiple pathophysiological domains of interest in AD. In examining both technical reliability and biotemporal stability in CSF (**S2 Fig**), we established baseline variability in analyte measures, a prerequisite for the design and interpretation of biomarker measures in studies of differential diagnosis, disease staging, and tracking of longitudinal change with disease progression or intervention response.

Biotemporal stability is often overlooked as a measure of assay reliability, but is important as metabolic, vascular, inflammatory, and other markers may fluctuate or otherwise vary over time. Analyte levels might be affected by circadian or seasonal rhythms, diet, environmental stressors, and intercurrent health issues. Such factors may compromise the comparative utility of these biomarkers, underscoring the importance of determining intra-individual variation prior to their inclusion as biomarkers in longitudinal studies or clinical trials. Determining baseline fluctuation of analytes also has statistical implications for the interpretation of both cross-sectional and longitudinal data, and can inform study design and sample size necessary to reveal significant differences in the primary outcomes of clinical trials.

From our initial screening of 60 unique protein/molecular targets, we identified 32 promising markers that were measurable in CSF, passed our technical reliability performance criteria of better than 15% CV, and presented low intra-individual variation between repeat collected samples. The panel of high performing candidate analytes we identified represent important pathophysiological domains in AD—including neurodegeneration, inflammation/immune modulation, neurovascular injury, metabolism and oxidative stress—allowing for multi-pathway profiling of disease state. Their performance criteria would qualify these assays in research, though better performance would be necessary for clinical use. In addition, the multiplexed nature of these assays requires that more testing be performed if they are to be used in long-term studies, when different "lots" of assay kits might be used over time. Multiplexed assays are more subject to lot to lot variability, cross reactivity issues, and matrix effects than single-plex assays, and as a result should be assessed for spike recovery and parallelism on a lot-to-lot basis [50].

Each of the core AD CSF biomarkers tested (tTau, Aβ_1–38_, Aβ_1–40_, and Aβ_1–42_) performed well in every measure, meeting the fit-for-purpose standards of sensitivity and stability for the MSD assays. These results were in agreement with published data evaluating the analytic performance of these proteins on this platform [51]. Of the non-core analytes, the non-Aβ and non-tau neurodegeneration markers NfL and FABP3 were also remarkably stable over a two-month interval, with comparable analytic performance to the classical AD markers. NfL is considered to be a marker of damage to large myelinated axons, and FABP3 is an abundant cytoplasmic protein that is thought to participate in the uptake, intracellular metabolism, and/or transport of long-chain fatty acids, playing a role in composition of lipid membranes [52–54]. NfL and FABP3 have both been reported to correlate with tTau [18,55] and/or Aβ [56]. However, neither is thought to be disease specific, but rather general markers of neurodegeneration [54,57–59]. They may be useful as complementary biomarkers for staging AD or perhaps quantifying the amount or degree of active neurodegeneration at the time of the sample.

Abnormalities in inflammatory cytokines, chemokines, and immune modulators have been reported in AD, but replications have been less consistent. Among the more robust has been the secreted glycoprotein YKL-40 [18,60–63], while data are less consistent on others such as monocyte chemokine MCP-1 [64,65] and proinflammatory cytokine IL-6 [66]. The value of these inflammatory analytes as biomarkers in the diagnosis, prognosis, or progression of AD/ADRD remains to be seen, but we can confirm the reliability and stability of their measurement for this purpose. In addition, we found that seven other inflammatory/immune molecules, not heretofore reported in CSF, also demonstrated good performance characteristics, including the interleukins IL-7, IL-12/23p40, IL-15, IL-16, and IL-8, MDC and MIP-1β.

The role of neurovascular injury has been of increasing interest in AD, but relevant biomarkers are not well established. Some of the more commonly reported include adhesion molecules ICAM-1 and VCAM-1 [67,68]. We identified eleven potential biomarkers (**Table 2**) of relevance to neurovascular injury that were reliably measurable in CSF and exhibited good biotemporal stability. Having established a method of reliably replicating the measurement of these markers will assist future studies investigating their roles in the pathogenesis of AD/ADRDs.

Many markers of metabolism and oxidative stress are reported to be present at very low concentrations in CSF compared to plasma or serum [69–71]. We were especially interested in measuring insulin levels in CSF, as insulin resistance is of great current interest in the field [7,72] and insulin levels have previously been reported to be altered in the CSF of AD patients [73]. Using MSD and three additional commercial assays for insulin we found very low concentrations in CSF, at or below the lower limits of detection. Further work, perhaps with new, ultrasensitive single molecule array assays will be needed to reliably measure this important metabolic hormone in CSF for AD studies.

Of the initial ten metabolism/oxidative stress biomarkers we surveyed, only five were reliably measurable in CSF, including sIR, adipokines adiponectin and leptin, the brain specific cholesterol metabolite 24-OHC, and oxidative stress related DNA modification 8-OHdG. Abnormalities in essential metabolic pathways, including trophic and metabolic signaling and regulation and cholesterol trafficking and turnover, have been reported in AD and associated with disease severity [74–78]. The relatively few reports prompt interest, and we hope the validation data we provide enable further study in this important area.

In conclusion, we have identified a pathophysiologically diverse set of CSF biomarkers that demonstrate consistent, reliable, and biotemporally stable quantification, establishing their potential for use in exploratory studies of AD. Subsequent work will assess the value of these high performing assays for inclusion in a practical biomarker panel for multi-dimensional molecular profiling in dementia as a tool for diagnosis and staging as well as assessing disease mechanisms, novel therapeutic target engagement of drugs or other biological interventions, and response in clinical trials. We conjecture that analyte profiles will differ among patients to varying degrees, thus allowing a personalized profile that may suggest a personalized treatment or prevention strategy.

## Supporting information

S1 Fig Assay sensitivity performance for CSF collected from subjects with MCI or mild dementia due to AD. (PDF)

S2 Fig Promising candidates that exhibited good technical replicability and biotemporal stability. (PDF)

S1 Table Descriptive statistics of study samples. (XLSX)

S2 Table Inter-assay reliability characteristics of biomarkers reliably measurable in CSF. (XLSX)

S3 Table Raw data for all analytes included in the study. (XLSX)
