# Supplementary material for: The technical reliability and biotemporal stability of cerebrospinal fluid biomarkers for profiling multiple pathophysiologies in Alzheimer’s disease
Source: PLoS One. 2018 Mar 5;13(3):e0193707. doi: 10.1371/journal.pone.0193707 (PMC5837100; doi:10.1371/journal.pone.0193707)
Supplement: S1 Fig — (PDF) [file pone.0193707.s001.pdf]

S1 Figure. Assay sensitivity performance for CSF collected from subjects with MCI or mild dementia due to AD.

Range of analyte concentrations in CSF and assay lower limit of detection (LLOD) for **(a)** core AD biomarkers, **(b)** Aβ and tau-independent markers of neurodegeneration, **(c)** metabolic and oxidative stress markers, **(d)** markers of inflammation and immune modulation, and **(e)** vascular injury markers. Red bars indicate mean LLOD value across three experimental blocks. Boxplots illustrate the median, inter-quartile range (25-75 percentile), and range (whiskers) of all measured samples. Boxplots in grey denote analytes that were not measurable in > 80% of samples, failing to meet cut-off criteria. Plotted using a log scale and converted all units to pg/mL. Abbreviations: ND, not detectable (indicates analytes where all sample measurements fell below the zero calibrator and concentration values could not be calculated).

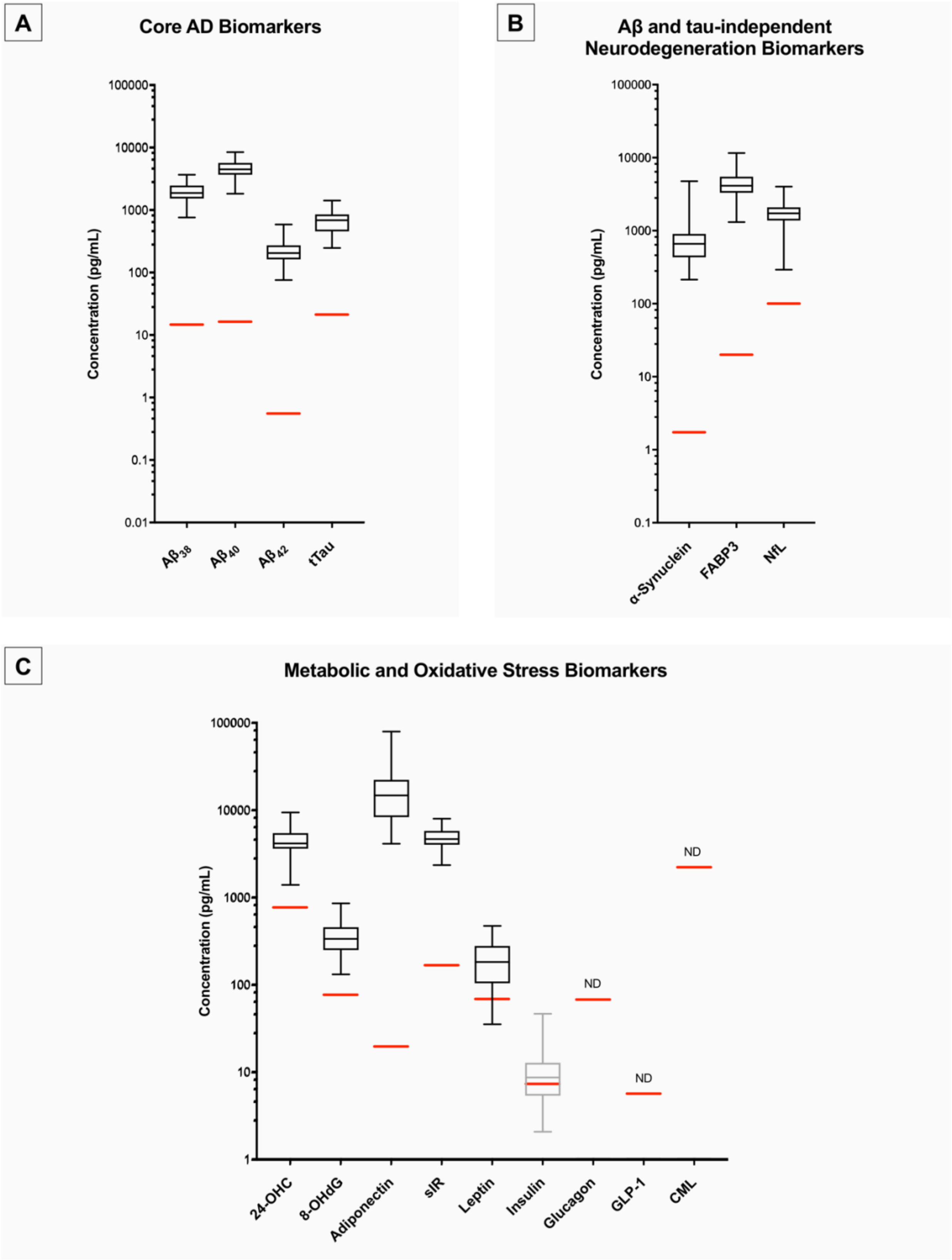
A
Core AD Biomarkers
100000
10000
1000
100
10
1
0.1
0.01
Concentration (pg/mL)
Aβ38
Aβ40
Aβ42
tTau
B
Aβ and tau-independent
Neurodegeneration Biomarkers
100000
10000
1000
100
10
1
0.1
Concentration (pg/mL)
α-Synuclein
FABP3
NfL
C
Metabolic and Oxidative Stress Biomarkers
100000
10000
1000
100
10
1
Concentration (pg/mL)
ND
ND
ND
24-OHC
8-OHdG
Adiponectin
sIR
Leptin
Insulin
Glucagon
GLP-1
CML

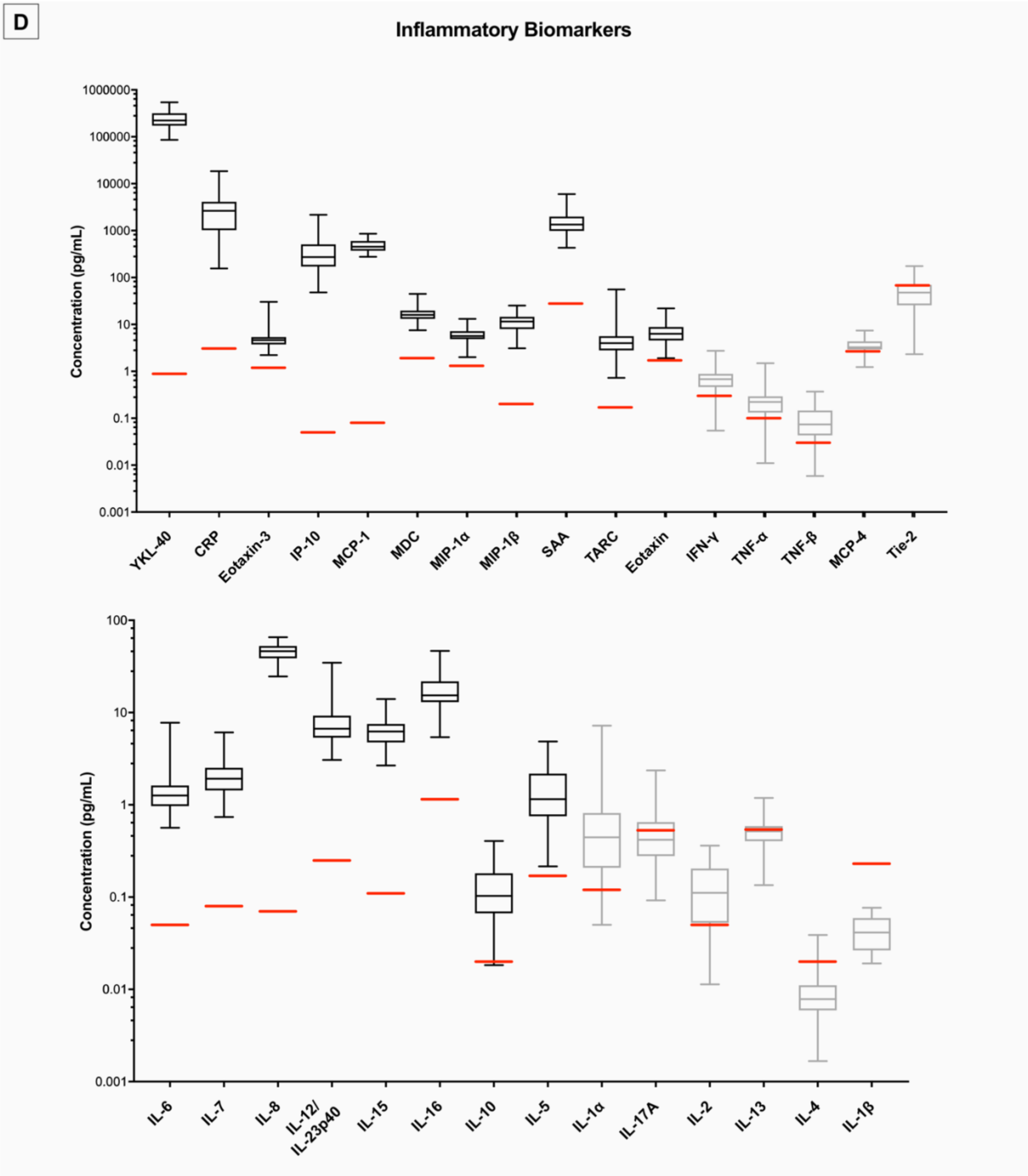
D
Inflammatory Biomarkers
Concentration (pg/mL)
1000000
100000
10000
1000
100
10
1
0.1
0.01
0.001
YKL-40
CRP
Eotaxin-3
IP-10
MCP-1
MDC
MIP-1α
MIP-1β
SAA
TARC
Eotaxin
IFN-γ
TNF-α
TNF-β
MCP-4
Tie-2
Concentration (pg/mL)
100
10
1
0.1
0.01
0.001
IL-6
IL-7
IL-8
IL-12/
IL-23p40
IL-15
IL-16
IL-10
IL-5
IL-1α
IL-17A
IL-2
IL-13
IL-4
IL-1β

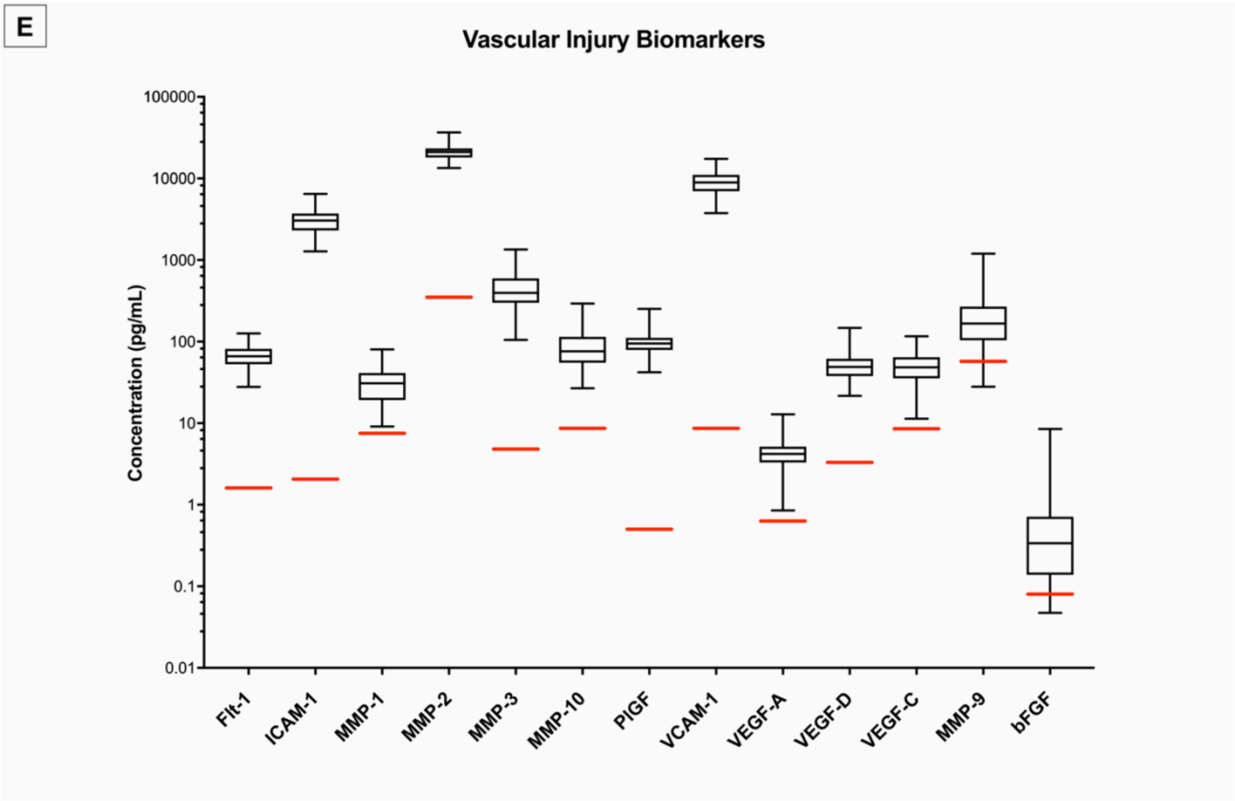

E
Vascular Injury Biomarkers
Concentration (pg/mL)
100000
10000
1000
100
10
1
0.1
0.01
Flt-1
ICAM-1
MMP-1
MMP-2
MMP-3
MMP-10
PIGF
VCAM-1
VEGF-A
VEGF-D
VEGF-C
MMP-9
bFGF
