# Supplementary material for: The technical reliability and biotemporal stability of cerebrospinal fluid biomarkers for profiling multiple pathophysiologies in Alzheimer’s disease
Source: PLoS One. 2018 Mar 5;13(3):e0193707. doi: 10.1371/journal.pone.0193707 (PMC5837100; doi:10.1371/journal.pone.0193707)
Supplement: S2 Fig — (PDF) [file pone.0193707.s002.pdf]

S2 Figure. Promising candidates that exhibited good technical replicability and biotemporal stability.

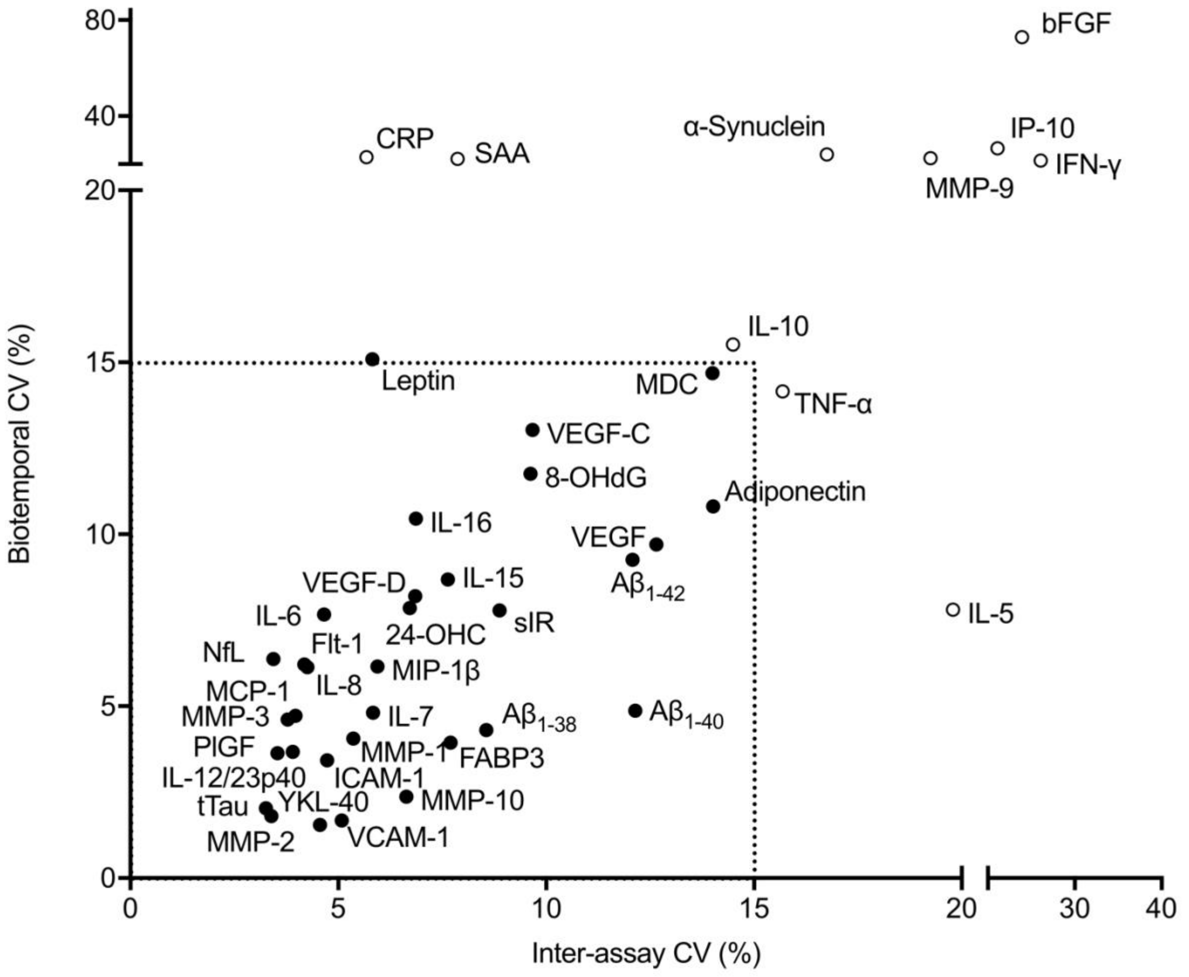

Fit-for-purpose criteria set at 15% for both inter-assay replicability and biotemporal variation. Of the 42 candidates that passed assay sensitivity and technical precision measures, 32 analytes were identified as promising candidates with acceptable inter-assay replicability and biotemporal stability (indicated with filled-in data points). Abbreviations: CV, coefficient of variation.
